# Development of a Method for Blocking Polysodiumoxy(methyl)siloxane Obtained in an Alcohol Medium

**DOI:** 10.3390/polym17152023

**Published:** 2025-07-24

**Authors:** Marina A. Obrezkova, Alina A. Nesterkina, Aziz M. Muzafarov

**Affiliations:** 1 Enikolopov Institute of Synthetic Polymeric Materials, Russian Academy of Sciences (ISPM RAS), Profsoyuznaya Str. 70, Moscow 117393, Russia; a.nesterkina@ispm.ru (A.A.N.); aziz@ispm.ru (A.M.M.); 2 Nesmeyanov Institute of Organoelement Compounds (NIOC RAS), Vavilova Str. 28, str. 1, Moscow 119334, Russia

**Keywords:** hydrolytic polycondensation, alkoxysodium salts, monosodiumoxy(methyl)(diethoxy)silane, polysodiumoxy(methyl)siloxane, polyfunctional matrix, blocking process, protective group

## Abstract

Polysodiumoxy(methyl)siloxane is a highly functional polymer matrix that can be used for the preparation of both functional and non-functional polymers, including molecular brushes. To determine the molecular weight parameters of the matrix, as well as its chemical structure, it is necessary to develop an effective method of blocking functional (in our case, sodiumoxy) groups due to their high reactivity. At the same time, the blocking product should represent a complete non-functionalized replica of polysodiumoxy(methyl)siloxane. Since the obtained polysodiumoxy(methyl)siloxane can contain both sodium- and hydroxy groups in its composition, the presence of both types of functional groups should be considered in the blocking process. In this work, we investigated the blocking process of polysodiumoxy(methyl)siloxane and the influence of blocking conditions on the blocked product. We carried out several variants of blocking, which differed in the order and method of introduction of reagents, as well as in the temperature regime. The chemical structure and molecular weight characteristics of the obtained polymers were analyzed by ^1^H NMR spectroscopy and gel permeation chromatography (GPC), respectively. According to the blocking results, only in one case, complete non-functionalized replicas of polysodiumoxy(methyl)siloxane were obtained, which allows this technique to be used as a tool for the analysis of complex, highly functionalized organosilicon systems.

## 1. Introduction

Organosilicon materials are used in various fields such as paint [1,2,3] and textile industries [4,5,6], automotive [7,8,9,10,11], aerospace [12,13,14,15], construction industry [16,17,18,19], cosmetology [20,21,22,23], bioengineering and medicine [24,25,26,27,28,29,30], etc.

Blocking reactions play an important role in the chemistry of organosilicon compounds, both for the determination of chemical structure and molecular weight parameters [31,32,33] and for a number of synthetic procedures in which the blocking group acts as a protective group [34,35]. Depending on the type of the blocking group [36,37], various blocking agents, such as triorganochlorosilanes [38], triorganosilanols [39], hexamethyldisiloxane [40], etc., are used. It is worth noting that after the blocking reaction, the reaction product should be a non-functionalized replica of the blocked compound.

There are different variants of the blocking reaction, which differ in the sequence of introduction of the blocking agent and the blocked compound [41,42]. For example, to estimate the amount of silanol groups (Si-OH) in the polycondensation (PC) product of diethoxydimethylsilane (DEDMS), their blocking with vinyldimethylchlorosilane (VDMCS) was carried out [43]. In the considered example, a solution of polyphenyl(hydroxy)siloxane in dry toluene was coupled with a solution of trimethylchlorosilane (TMCS) and pyridine. The obligatory requirement for blocking was observance of conditions excluding the possibility of homocondensation of silanol groups (Si-OH) and subsequent change of the initial composition of reaction products. Completeness of blocking was controlled by IR spectroscopy by disappearance of absorption bands in the region 3600–3800 cm^−1^ corresponding to valence vibrations of OH-groups. End silanol groups were counted by ^1^H NMR spectroscopy by comparing the integral intensities of the signals of vinyl groups in the blocked group and methylsilyl groups [44]. To use this method, the signals of the protons of organic substituents in the blocking group and the main chain must be confidently detectable in terms of chemical shift. This is the prerequisite for the choice of a blocking agent.

In the case of blocking sodium dimethylsiloxanediolates with VDMCS, the same sequence of introducing the blocking agent and the compound to be blocked was used [42]. The only difference is that the blocking functional groups are sodiumoxy groups.

Monosodiumoxy(organo)(alkoxy)silanes have great potential for the synthesis of organosilicon compounds with various structures [45]. These are organosilicon monomers containing different functional groups at the silicon atom, which differ in their chemical nature [46].

Successful realization of chemical transformations of monosodiumoxy(organo)(alkoxy)silanes with the participation of only alkoxy groups led to the formation of linear polyorganosiloxanes containing sodiumoxy groups at each silicon atom [44]. This approach allowed the preparation of linear siloxane polymer matrices capable of further transformations [47]. Polysodiumoxy(methyl)siloxanes are polymeric compounds of ionomeric nature. Their stability is due to the uniform charge distribution along the molecular chain. Their unique difference from carboxy-chain ionomers lies in the fact that two mutually exclusive elements of the structure are linked in a single origin: silanolate groups and siloxane chain. Any deviation from stoichiometry exceeding a few mole percent will lead to partial or complete cleavage of the molecular structure into separate fragments. At the same time, they cannot be called metastable. In the absence of moisture and other hydroxyl-containing or acidic impurities, they are stable and can be stored for years without changes. It is more correct to consider them as highly reactive polyfunctional polymers.

As for most of the highly functionalized compounds [37], an efficient analysis system was needed to evaluate the chemical structure and molecular weight parameters, as well as to select the optimal conditions for the synthesis of linear polysodiumoxy(methyl)siloxane matrix. Spectral analysis of functional compounds, especially considering the high reactivity of all groups, was difficult and did not guarantee qualitative results, and chromatographic methods of investigation were completely excluded due to the high reactivity of numerous functional groups.

None of the above approaches to blocking was suitable for blocking a new complex polyfunctional polymer system. And if in the considered examples, small changes in methods and approaches allowed for achieving the desired result; in this case, the task was much more complicated: Firstly, it was necessary to block not the final product of the reaction but a semi-product—the basis for subsequent transformations; secondly, the object of study contained not one and not two types of functional groups but three; thirdly, it is necessary to take into account that the object is not soluble in organic solvents acceptable for these reagents; fourth, the blocking operation had not only analytical but also preparative character, as we had to determine not only the structure of the product but also to work it up by the simplest, most efficient, and most error-free preparative method. Therefore, the development of an approach that can be used to reliably analyze complex functionalized organosilicon systems (including multi-arm star-shaped polymers [48], nanogels, and complex hyperbranched architectures [49,50]) seems to us to be an urgent task.

Thus, the task of this study was to compare the three closest variants of blocking, reflecting the composition and structure of the target product, and to choose one that allows solving not only the analytical part of the problem but also the preparative one.

## 2. Materials and Methods

### 2.1. Materials

Commercial reagents, sodium hydroxide (“Spectrchim”, Saint Petersburg, Russia), sodium sulfate (“Component-Reaktiv”, Moscow, Russia), and VDMCS (98%, “ABCR”, Karlsruhe, Germany) were used without further purification. Methyltriethoxysilane (“Penta-91”, Shchyolkovo, Russia), pyridine, hexane, methanol, ethanol, propan-2-ol, and n-butanol (“SpektrChem”, Saint Petersburg, Russia) were pretreated according to generally accepted methods [51,52]. All solvents used in this work were dried by distillation over hydride in the presence of argon. After drying, the solvents were stored over 3A molecular sieves. Methyltriethoxysilane was purified by distillation. The synthesis of monosodiumoxy(methyl)(diethoxy)silane was carried out according to the method [46].

### 2.2. Methods

GPC analysis was performed on a chromatographic system: high-pressure pump LC-10ADvp (Shimadzu, Kyoto, Japan), a refractometer detector Smartline RI 2300 (KNAUER, Berlin, Germany), and thermostat JETSTREAM 2 PLUS (KNAUER, Berlin, Germany). The temperature was 40 ± 0.1 °C, the eluent was toluene + 2% THF, and the flow rate was 1.0 mL/min. Columns 300 × 7.8 mm, Phenogel sorbent (Phenomenex, Torrance, CA, USA), 5 μm, pore size from 50 Å to 10^5^ Å. Calibration of columns relative to Agilent polystyrene standards (USA). Processing of chromatograms and calculation of molecular weight parameters are given by the Multichrome for Windows program, version 1.6 (Ampersend, Moscow, Russia).

^1^H NMR spectra were recorded on a Bruker WP 250 SY spectrometer (Bruker Corporation, Berlin, Germany). The solvent was CDCl_3_. Spectra processing program “ACD LABS”.

The GPC and NMR spectroscopic analyses were performed in the collaborative access center “Center for Polymer Research” of ISPM RAS.

### 2.3. Synthesis of Polysodiumoxy(methyl)siloxane

Synthesis of polysodiumoxy(methyl)siloxane using the example of hydrolytic polycondensation reaction (HPC) at a monomer concentration of 10% in methanol solution. Polysodiumoxy(methyl)siloxanes were prepared by HPC in organic solvents such as methanol, ethanol, propan-2-ol, and n-butanol at a monomer concentration of 10% and in propan-2-ol at a monomer concentration of 30% at room temperature (25 °C). In a one-neck round bottom flask equipped with a magnetic stirrer and a dropping funnel, 114 mL of dry methanol and 10.00 g (0.058 mol) of monosodiumoxy(methyl)(diethoxy)silane were loaded under an argon cushion. After dissolution of monosodiumoxy(methyl)(diethoxy)silane and vigorous stirring, 1.0 g (0.058 mol) of water was added. At the end of the reaction (5 h), the solvent was removed by an oil pump (1 Torr). A powdery product of white color was obtained. The yield of the reaction product was 97%.

### 2.4. Blocking of Polysodiumoxy(methyl)siloxane Under Blocking Conditions 1

In a three-neck round bottom flask equipped with a magnetic stirrer, a reflux condenser, a thermometer, and a dropping funnel, 5 g (0.0510 mol) of polysodiumoxy(methyl)siloxane, 18 mL of hexane, and 4.51 mL (0.0561 mol) of pyridine were loaded under an argon cushion. Upon cooling to −70 °C and vigorous stirring, 7.67 mL (0.0561 mol) of vinyldimethylchlorosilane was added. The mixture was stirred at room temperature (25 °C) for 2–3 h. The mixture was then washed with water in hexane to neutral medium, and then the residual water was removed over anhydrous sodium sulfate for 24 h. The precipitate was filtered off. The solvent was distilled off under vacuum (1 Torr). The obtained product was a transparent, colorless, viscous liquid. The yield of the product was 81%. ^1^H NMR (CDCl_3_): δH: 5.69–6.21 ppm (3H; SiCH=CH_2_); 0.12–0.19 ppm (6H; SiCH_3_); 0.02–0.12 ppm (3H; SiCH_3_).

### 2.5. Blocking of Polysodiumoxy(methyl)siloxane Under Blocking Conditions 2

In a three-neck round bottom flask equipped with a magnetic stirrer, a reflux condenser, a thermometer, and a dropping funnel, 5 g (0.0510 mol) of polysodiumoxy(methyl)siloxane in 18 mL of hexane at −70 °C was loaded under argon cushion, and 7.67 mL (0.0561 mol) of vinyldimethylchlorosilane was added under vigorous stirring. The mixture was stirred at room temperature (25 °C) for 2–3 h. Following the formation of an acidic medium, the mixture was washed with water, and 0.41 mL (0.0051 mol) of pyridine was added; then, the residual water was removed over anhydrous sodium sulfate for 24 h. The precipitate was filtered off. The solvent was removed by an oil pump (1 Torr). The obtained product was a transparent, colorless, viscous liquid. The yield of the product was 75%. ^1^H NMR (CDCl_3_): δH: 5.69–6.21 ppm (3H; SiCH=CH_2_); 0.12–0.19 ppm (6H; SiCH_3_); 0.02–0.12 ppm (3H; SiCH_3_).

### 2.6. Blocking of Polysodiumoxy(methyl)siloxane Under Blocking Conditions 3

In a three-neck round bottom flask equipped with a magnetic stirrer, a reflux condenser, a thermometer, and a dropping funnel, 8.44 mL (0.0561 mol) of vinyldimethylchlorosilane, 10 mL of hexane, and 4.51 mL (0.0561 mol) of pyridine were loaded under an argon cushion. Upon cooling to −70 °C and vigorous stirring, 5 g (0.0510 mol) of polysodiumoxy(methyl)siloxane in 18 mL of hexane was added dropwise. The mixture was stirred at room temperature (25 °C) for 2–3 h. The mixture was then washed with water in hexane to neutral medium, and then the residual water was removed over anhydrous sodium sulfate for 24 h. The precipitate was filtered off. The solvent was removed by an oil pump (1 Torr). The obtained product was a transparent, colorless, viscous liquid. The yield of the product was 79%. ^1^H NMR (CDCl_3_): δH: 5.69–6.21 ppm (3H; SiCH=CH_2_); 0.12–0.19 ppm (6H; SiCH_3_); 0.02–0.12 ppm (3H; SiCH_3_).

### 2.7. Blocking of Polysodiumoxy(methyl)siloxane Under Blocking Conditions 4

In a one-neck round bottom flask equipped with a magnetic stirrer and a dropping funnel, 5 g (0.0510 mol) of polysodiumoxy( methyl)siloxane in 18 mL of hexane was loaded under an argon cushion. With vigorous stirring, 7.67 mL (0.0561 mol) of vinyldimethylchlorosilane in 5 mL of hexane was added for over two hours. The mixture was stirred at room temperature (25 °C) for 2–3 h. Following the formation of an acidic medium, the mixture was washed with water and 0.41 mL (0.0051 mol) of pyridine, and then the residual water was removed over anhydrous sodium sulfate for 24 h. The precipitate was filtered off. The solvent was removed by an oil pump (1 Torr). The obtained product was a clear, colorless, viscous liquid. The yield of the product amounted to 43%. ^1^H NMR (CDCl_3_): δH: 5.69–6.21 ppm (3H; SiCH=CH_2_); 0.12–0.19 ppm (6H; SiCH_3_); 0.02–0.12 ppm (3H; SiCH_3_).

## 3. Results and Discussion

In a number of applications, such as the synthesis of dense molecular brushes, polysodiumoxy(methyl)siloxanes are very promising as highly functionalized matrices due to their linear structure and high reactivity of the sodiumoxy groups. But when improperly treated, these advantages become a major cause of failure. In our early work, success was achieved by a complicated synthesis with the slow introduction of water into an excess of the alkoxysodium salt of methylsiloxane with simultaneous removal of the resulting alcohol [44]. At the transition to very promising alcoholic media, the used chemical technique not only did not guarantee success but actually completely excluded it. Therefore, the development of a new simplified “alcohol” approach to the synthesis of polysodiumoxy(methyl)siloxanes begins with the development of a method for their blocking, the conditions of which can be further used to obtain the target polymer systems.

**Figure 1:**
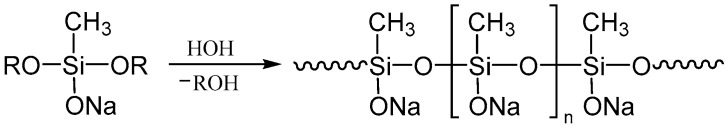
The scheme of the HPC reaction of monosodiumoxy(methyl)(diethoxy)silane.

Polysodiumoxy(methyl)siloxanes were prepared by HPC in organic solvents, such as methanol, ethanol, propan-2-ol, and n-butanol, at a monomer concentration of 10% and in propan-2-ol, at a monomer concentration of 30% at room temperature (Figure 1). In all cases, a stoichiometric amount of water was used. At the end of the HPC, the solvent was removed using an oil pump. As a result, polysodiumoxy(methyl)siloxanes were obtained as white powders.

Since the obtained polysodium salts are insoluble in all solvents, we used their suspensions in toluene or hexane.

As it was previously mentioned [42,43], to evaluate the chemical structure of functional polymethylsiloxanes, it is better to choose a blocking agent with different organic substituents from methyl substituents. Therefore, VDMCS was chosen as a blocking agent to carry out the blocking reaction of sodiumoxy groups in the obtained polysodiumoxy(methyl)siloxanes. It is known [41] that the blocking reaction should be carried out in nonpolar organic solvents (n-alkanes, aromatic hydrocarbons, etc.), in some cases allowing short-term heating of the reaction mixture at 35–65 °C. The use of polar solvents, such as sulfuric ether, at elevated temperatures can lead to a sharp activation of side processes and, ultimately, to a complex, difficult-to-separate mixture of products [41]. To avoid distortions in the structure of the analyzed compounds during the trialkylsilylation reaction, triorganochlorosilane should be used in small excess, which can then be easily neutralized. Such conditions allowed the prevention of the formation of by-products not typical of the main reaction.

**Figure 2:**
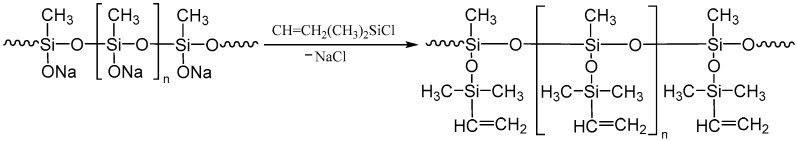
The scheme for the blocking reaction of polysodiumoxy(methyl)siloxane with vinyldimethylchlorosilane.

**Figure 3:**
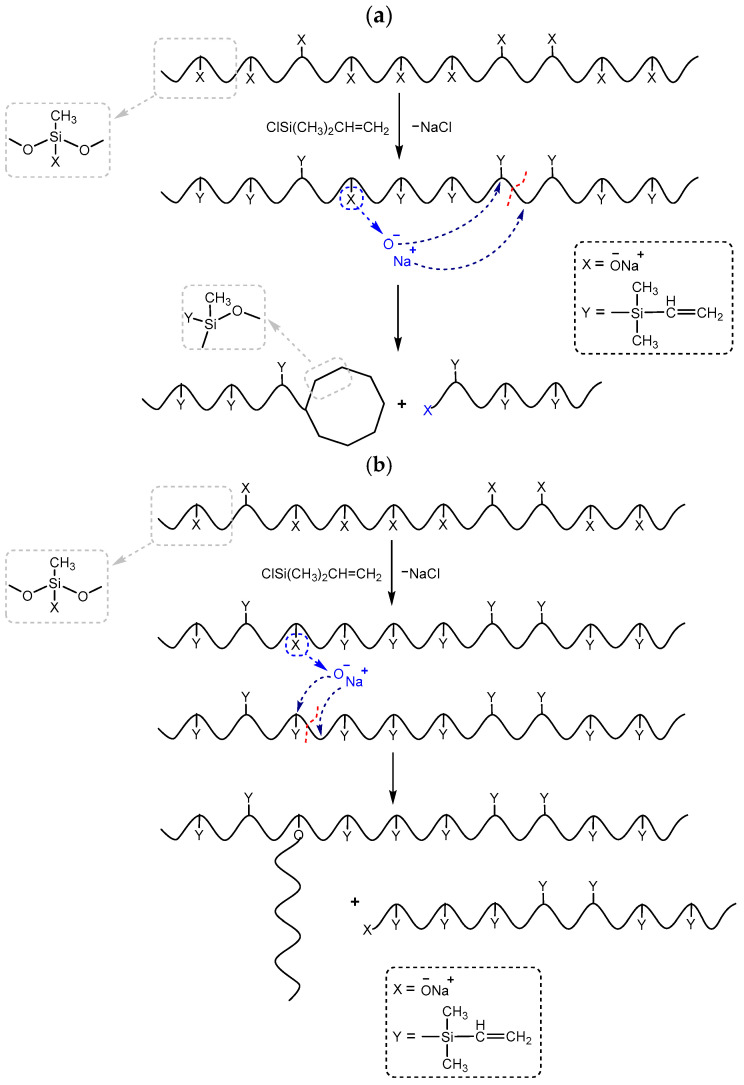
Schemes of possible side reactions resulting from incomplete blocking of hydroxyl groups in polysodium salt where (**a**) intramolecular interactions, (**b**) intermolecular interactions. The red dotted line indicates the cleavage of the siloxane bond.

In general terms, the blocking response can be represented as follows (Figure 2):

As described above, the resulting polysodiumoxy(methyl)siloxane may contain both sodiumoxy and hydroxy groups in its composition, depending on the conditions of HPC. Partial preservation of sodiumoxy groups in the blocked variant of polysodiumoxy(methyl)siloxane can lead to cleavage of the siloxane bond of the blocked product [53,54,55,56] (Figure 3). Such a gap can lead to both intramolecular interactions (Figure 3a) and intermolecular interactions (Figure 3b).

**Figure 4:**
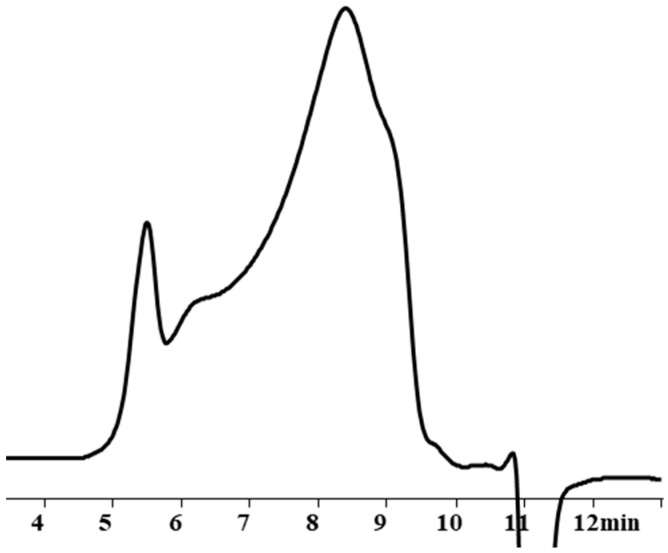
GPC curves of blocked replicas of polysodiumoxy(methyl)siloxanes blocked under blocking conditions 4.

**Figure 5:**
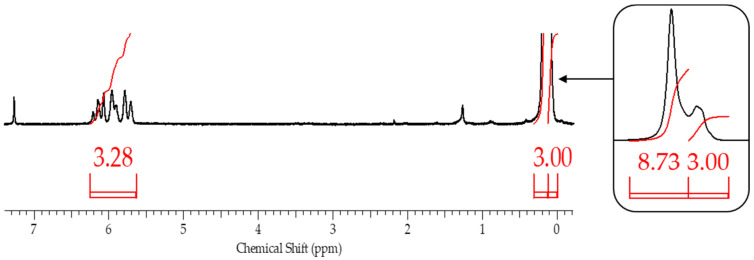
^1^H NMR spectra of blocked replicas of polysodiumoxy(methyl)siloxanes blocked under blocking conditions 4.

This assumption, indicating the occurrence of side processes, was artificially modeled when carrying out the blocking reaction under blocking conditions 4. In this case, chlorosilane was added dropwise to polysodiumoxy(methyl)siloxane over two hours at room temperature, and pyridine was added immediately before washing. In such a blocking reaction, there was no instantaneous reaction of sodiumoxy groups with chlorosilane; i.e., there was a partial substitution of sodiumoxy groups by chlorosilyl groups, which led to a redistribution of the charge of the sodiumoxy groups that had not yet reacted, which now acted as agents that cleave the siloxane chain (Figure 3). This situation is aggravated by pyridine introduced before washing, which accelerates the cleavage of the siloxane bond due to charge separation by ion solvation. According to the GPC data, the product of such blocking had a broad molecular weight distribution (Figure 4, Table 1), and the ratio of signals on the ^1^H NMR spectrum differed from the theoretical one (Figure 5, Table 1).

**Figure 6:**
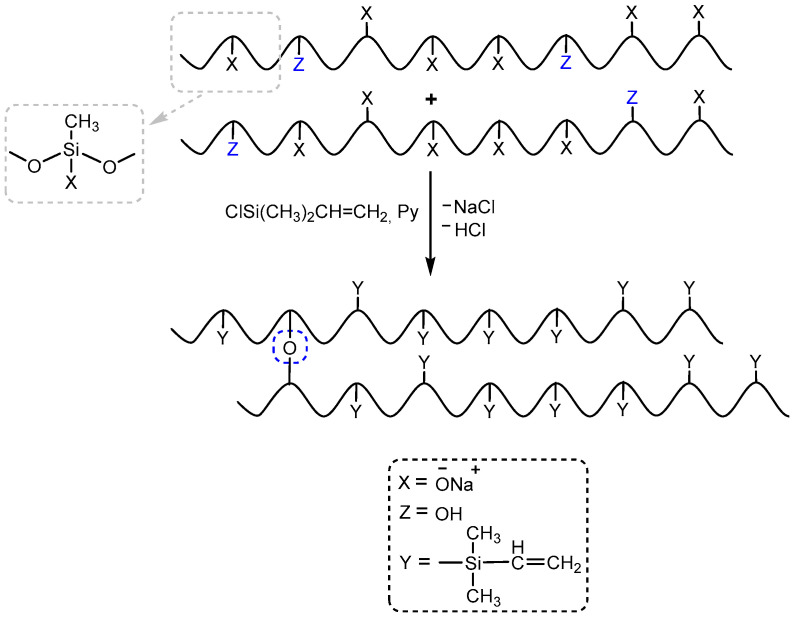
The schemes of possible side reactions occurring in the presence of hydroxyl groups in the polysodium salt (where Py—Pyridine).

The presence of hydroxyl groups in polysodiumoxy(methyl)siloxane during blocking with triorganochlorosilane can distort the structure of the blocking product in the form of their condensation in the presence of ionic impurities, which can be activated by pyridine (Figure 6).

**Figure 7:**
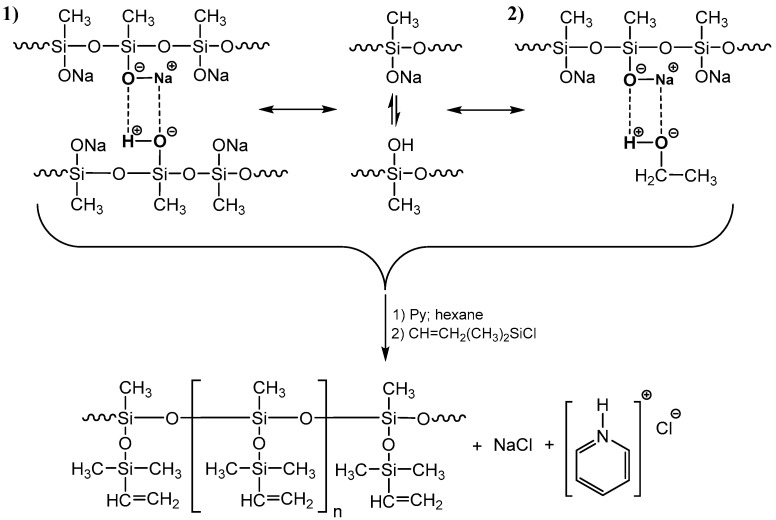
Scheme of stabilization of functional groups in the polysodiumoxy(methyl)siloxane (where Py—Pyridine).

Condensation of hydroxyl groups most likely occurs just in the blocking process (Figure 6) since, at low concentrations of hydroxysilyl groups, they are stabilized as a complex with sodiumoxy groups in the HPC process (Figure 7(1)).

With proper blocking, the decomposition and blocking of such a complex will not lead to chain distortions, as well as its alcoholic variant (Figure 7(2)). Actually, in the expectation of such stabilization of the alcoholic complex, alcohols were chosen as promising solvents for these systems.

Thus, both options can lead to distortion of the target structure during the blocking process. Therefore, a blocking variant should be chosen that considers the presence of both types of functional groups and their unambiguous blocking. That is, neutralization of sodiumoxy groups should be simultaneous, which will prevent cleavage of the product siloxane bonds, and blocking of hydroxyl groups should prevent their condensation, which may lead to the appearance of branching or cyclic fragments (Figure 6).

In order to exclude all possible deviations from the ideal linear structure of the investigated system in the process of blocking, we analyzed three variants of the reaction medium treatment (blocking conditions 1, blocking conditions 2, and blocking conditions 3). All three variants of blocking were carried out at the temperature of the reaction mixture of −70 °C, which was maintained until complete mixing (digging) of the “participants” of the blocking reaction. It was necessary to exclude the influence of mixture heating on the final reaction product. Pyridine was used as the hydrogen chloride acceptor in all cases. Hexane was chosen as the solvent of the blocking reaction. The difference in the blocking variants was only in the chosen sequence of addition of blocking agent, hydrogen chloride acceptor, and polysodiumoxy(methyl)siloxane. In all cases, the resulting reaction product was washed with distilled water in hexane to neutral medium, and the residual water was removed by drying over anhydrous sodium sulfate for 24 h. Then, the precipitate was filtered, the solvent was removed at a rotary evaporator, and traces of solvent were removed at an oil pump.

Under blocking conditions 1 and 2, chlorosilane was added dropwise to polysodiumoxy(methyl)siloxane. The only difference was that in blocking conditions 1, pyridine taken per all chlorosilane was added immediately to the reaction flask, whereas under blocking conditions 2, pyridine was taken per excess chlorosilane only and was added immediately before washing off the blocking product.

Under blocking conditions 3, the sequence of introduction of the blocking agent and polysodiumoxy(methyl)siloxane was reversed; that is, polysodiumoxy(methyl)siloxane was added to the VDMCS. Pyridine, as under blocking conditions 1, was calculated for the entire volume of chlorosilane.

**Figure 8:**
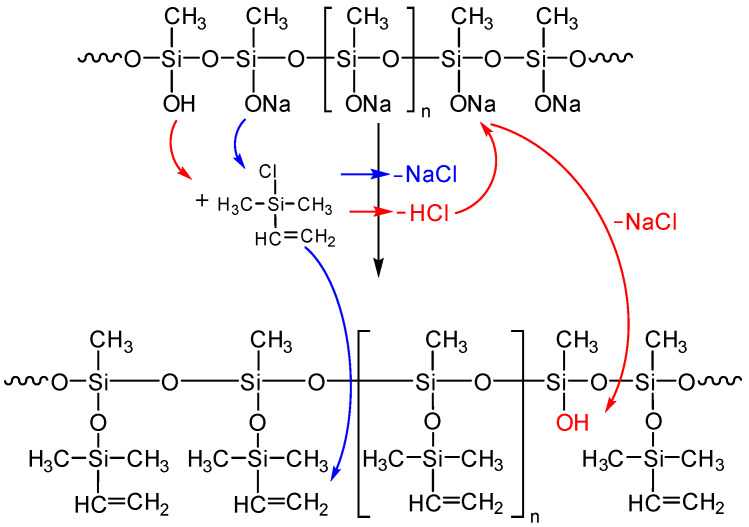
Competitive scheme of blocking of the sodiumoxy groups in the polysodiumoxy(methyl)siloxane by hydrogen chloride (blue color—target reaction, red color—side reaction).

The essence of the experimental differences lies in the unpredictability of the concentration of hydroxyl groups in the composition of the polymeric sodium salt. At this point, the heterogeneous reaction mass cannot be adequately analyzed in any way other than by blocking and subsequent analysis. The importance of this element of the technique lies in the fact that while the interaction of the sodiumoxy groups of the salt with triorganochlorosilane yields non-active sodium chloride, the interaction of the chlorosilane with the hydroxysilyl groups yields hydrogen chloride, which can act as a competitor to the blocking agent. In this case, a new hydroxyl group will appear on the silicon atom instead of the silanolate group, and the process will develop according to the Scheme in Figure 8.

To prevent this from happening, pyridine is introduced into the system as a hydrogen chloride acceptor, as demonstrated earlier in [43]. In turn, the amount and time of pyridine introduction can also be important. Therefore, these parameters were varied in the experiments.

**Figure 9:**
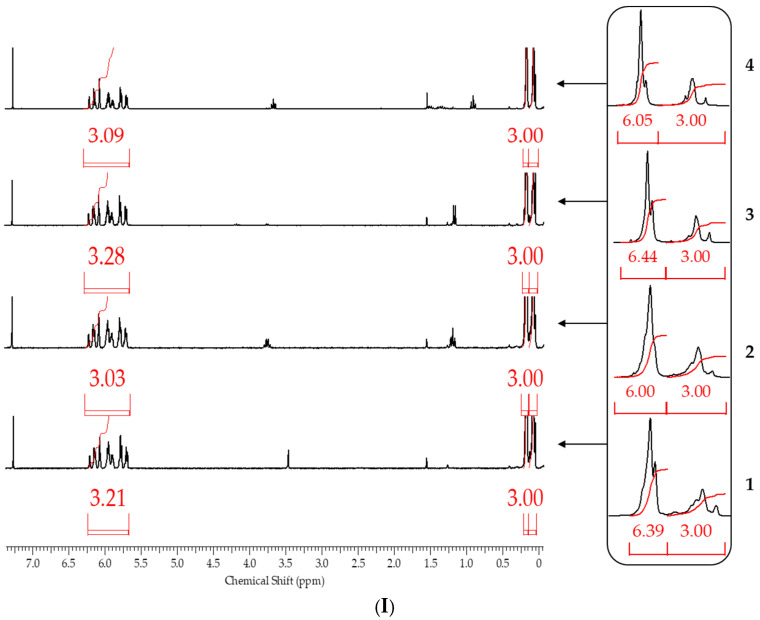

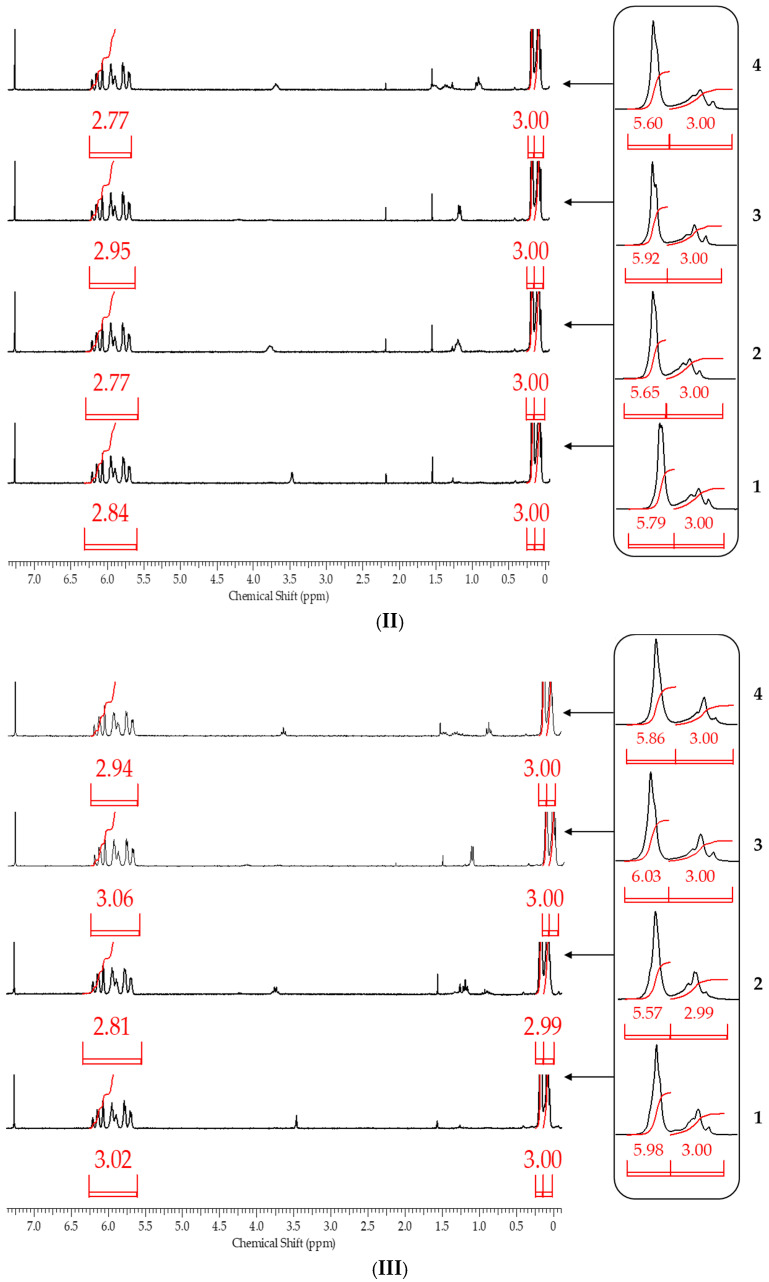
^1^H NMR spectra of polysodiumoxy(methyl)siloxanes obtained in (1) methanol, (2) ethanol, (3) propan-2-ol, (4) n-butanol, and blocked under blocking conditions: (**I**)—1, (**II**)—2, and (**III**)—3.

After blocking, the obtained polymers were analyzed by gel permeation chromatography (GPC) and ^1^H NMR spectroscopy to determine the molecular weight parameters and chemical structure, respectively. ^1^H NMR spectroscopy proved to be the most informative method (Figure 9).

From the ^1^H NMR spectra, the composition of the resulting polymer can be determined with accuracy within the error of the method by the ratio of the proton signals of the vinyl and methyl groups in the side substituent and the methyl group at the silicon atom in the main chain. The table below summarizes the blocking results (Table 2).

**Figure 10:**
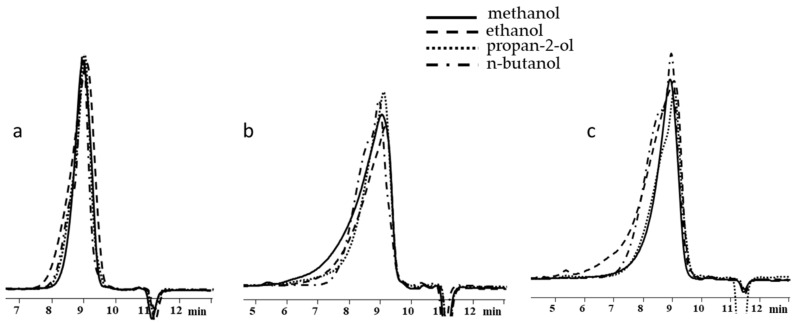
GPC curves of polysodiumoxy(methyl)siloxanes blocked under blocking conditions 1: (**a**) 1, (**b**) 2, and (**c**) 3. Toluene, 10^4^ Å.

It follows from the table that only when the blocking conditions of blocking conditions 1 (chlorosilane is added to the mixture of polysodiumoxy(methyl)siloxane, pyridine, and hexane) are met, there are no distortions in the structure of the formed reaction products, according to the ^1^H NMR spectroscopy data. At the other two variants of blocking, the underestimation of signals of protons of vinyl and methyl groups in the side substituent is observed, which may indicate the occurrence of side processes in the form of cleavage of the siloxane bond or condensation. The increase in molecular weight and polydispersity of the formed polymers can be used as a confirmation of the occurrence of side processes under blocking conditions 2 and 3 (Table 2, Figure 10).

Similar blocking studies were carried out for polysodiumoxy(methyl)siloxane prepared by the HPC reaction in propan-2-ol at a monomer concentration of 30%. Again, the same pattern was found in this case (Table 3).

**Figure 11:**
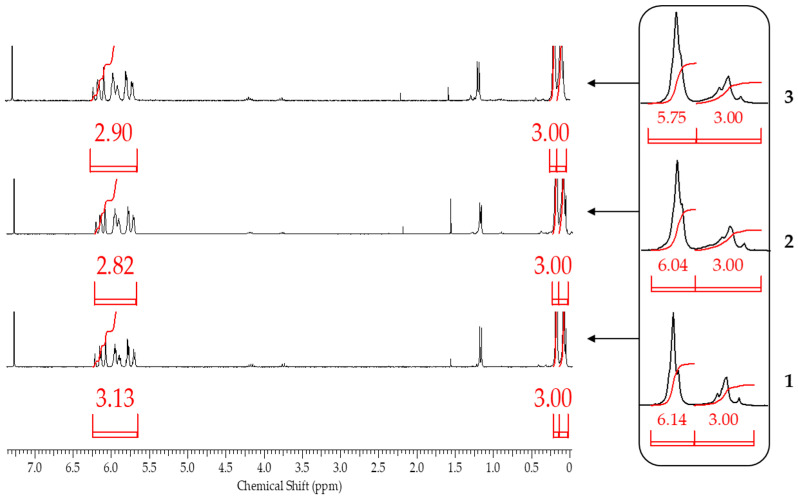
^1^H NMR spectra of polysodiumoxy(methyl)siloxanes obtained in propan-2-ol and blocked by the following blocking conditions: 1, 2, and 3.

According to ^1^H NMR spectroscopy data, only when blocking sodiumoxy groups in polysodiumoxy(methyl)siloxane under blocking conditions 1, a polymer was obtained in which the ratio of proton group signals did not contradict the theoretically calculated one (Figure 11).

**Figure 12:**
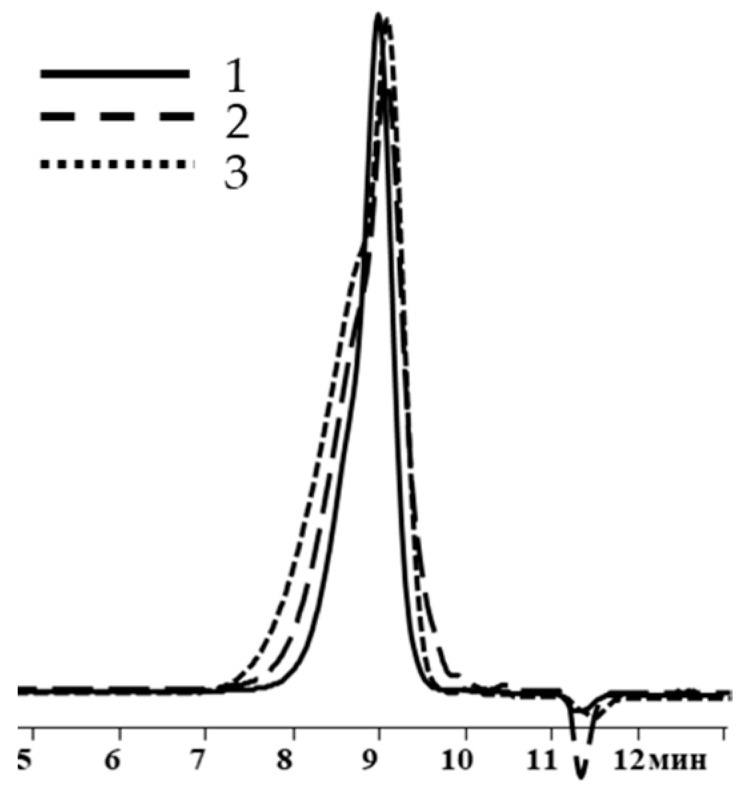
GPC curves of polysodiumoxy(methyl)siloxanes blocked under blocking conditions 1, 2, and 3.

As in previous cases, the increase in weight-average molecular weight and molecular weight distribution occurred when going from blocking conditions 1 to blocking conditions 2 and 3 (Table 3, Figure 12).

Thus, according to the ^1^H NMR and GPC data, method 1, i.e., when chlorosilane was added to the pyridine/polysodium salt mixture at −70 °C, was found to be an effective way to block the sodiumoxy groups in polysodiumoxy(methyl)siloxane.

## 4. Conclusions

A system of blocking (ratio and method of reagent introduction, temperature regime, and method of experimental data processing) of sodiumoxy groups in polysodiumoxy(methyl)siloxane by vinyldimethylchlorosilane has been developed. As a result of blocking, complete non-functionalized replicas of polysodiumoxy(methyl)siloxane were obtained, which allows us to use this technique as a tool for the analysis of complex, highly functionalized organosilicon systems, namely, for the characterization of their chemical structure and molecular weight characteristics. The improvement of the blocking method in the future will allow us to solve the problem of indistinguishability of end and side blocking groups, which is very important for understanding the structural heterogeneity of the studied systems. We hope to find a solution to this problem in our future studies by developing new selective blocking agents and sequences for their application.

## Figures and Tables

**Table 1:** Characterization of blocked replicas of polysodiumoxy(methyl)siloxanes under blocking conditions 4.

Blocking Conditions	Solvent	^1^H NMR H_Me_/H_(Me)2_/H_Vi_ 3:6:3	GPC, 10^4^ Å, Toluene		
			M_N_	M_W_	M_W_/M_N_
4	methanol	3:8.73:3.28	6700	54,900	8.24

**Table 2:** Characterization of blocked replicas of polysodiumoxy(methyl)siloxanes obtained at a monomer concentration of 10%.

Blocking Conditions	Solvent	^1^H NMR H_Me_/H_(Me)2_/H_Vi_ 3:6:3	GPC, 10^4^ Å, Toluene		
			M_N_	M_W_	M_W_/M_N_
1	methanol	3:6.39:3.21	2700	3000	1.14
	ethanol	3:6.00:3.03	2900	3900	1.32
	propan-2-ol	3:6.44:3.28	2900	3400	1.19
	n-butanol	3:6.05:3.09	3200	3900	1.20
2	methanol	3:5.79:2.84	3600	11,500	3.18
	ethanol	3:5.65:2.77	3500	11,100	3.14
	propan-2-ol	3:5.92:2.95	3500	8900	2.58
	n-butanol	3:5.60:2.77	3500	5100	1.47
3	methanol	3:5.98:3.02	3400	7800	2.30
	ethanol	3:5.57:2.81	4000	16,800	4.22
	propan-2-ol	3:6.03:3.06	3300	8000	2.41
	n-butanol	3:5.86:2.94	3900	6300	1.62

**Table 3:** Characterization of blocked replicas of polysodiumoxy(methyl)siloxanes obtained at a monomer concentration of 30%.

Blocking Conditions	^1^H NMR H_Me_/H_(Me)2_/H_Vi_ 3:6:3	GPC, 10^4^ Å, Toluene		
		M_N_	M_W_	M_W_/M_N_
1	3:6.14:3.13	2900	3500	1.21
2	3:6.04:2.82	2800	4000	1.41
3	3:5.75:2.90	3300	4900	1.46

## Data Availability

The original contributions presented in this study are included in the article/Supplementary Material. Further inquiries can be directed to the corresponding author.

## Supplementary Materials

The following supporting information can be downloaded at: https://www.mdpi.com/article/10.3390/polym17152023/s1, Figure S1. ^29^Si NMR spectra of polysodiumoxy(methyl)siloxanes obtained in ethanol and blocked under blocking conditions: I—1, II—2, III—3. Figure S2. IR spectra of polysodiumoxy(methyl)siloxanes obtained in ethanol and blocked under blocking conditions: I—1, II—2, III—3.

## Abbreviations

The following abbreviations are used in this manuscript:

GPC	Gel permeation chromatography
Si-OH	Silanol groups
PC	Polycondensation
DEDMS	Diethoxydimethylsilane
VDMCS	Vinyldimethylchlorosilane
TMCS	Trimethylchlorosilane
HPC	Hydrolytic polycondensation
