# Supplementary material for: Development of a Method for Blocking Polysodiumoxy(methyl)siloxane Obtained in an Alcohol Medium
Source: Polymers (Basel). 2025 Jul 24;17(15):2023. doi: 10.3390/polym17152023 (PMC12349120; doi:10.3390/polym17152023)
Supplement: Supplementary file 1 [file polymers-17-02023-s001.zip › polymers-3716044-supplementary.pdf]

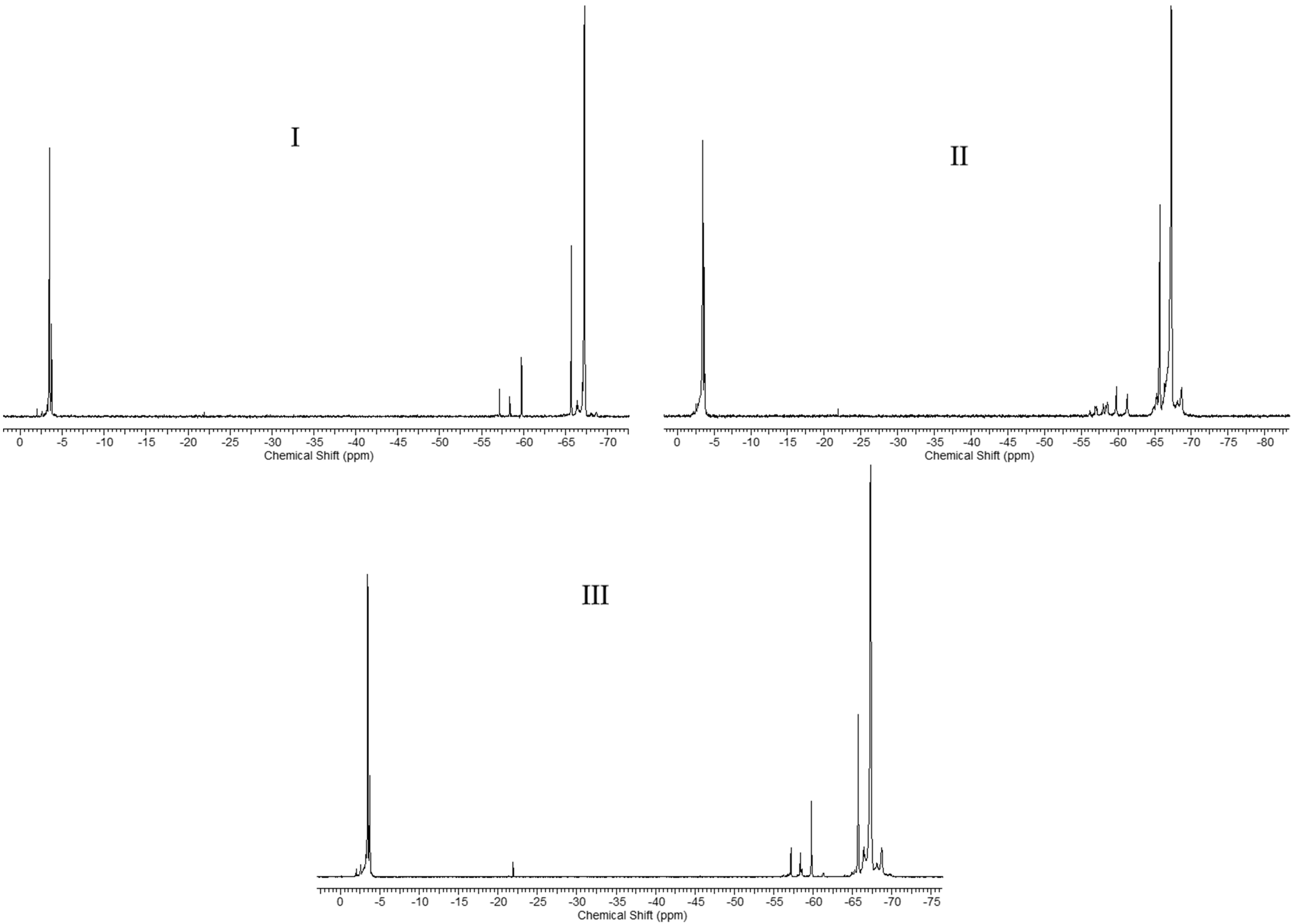

Figure S1. ^{29}Si NMR spectra of polysodiumoxy(methyl)siloxanes obtained in ethanol and blocked under blocking conditions: I - 1, II - 2, III – 3.

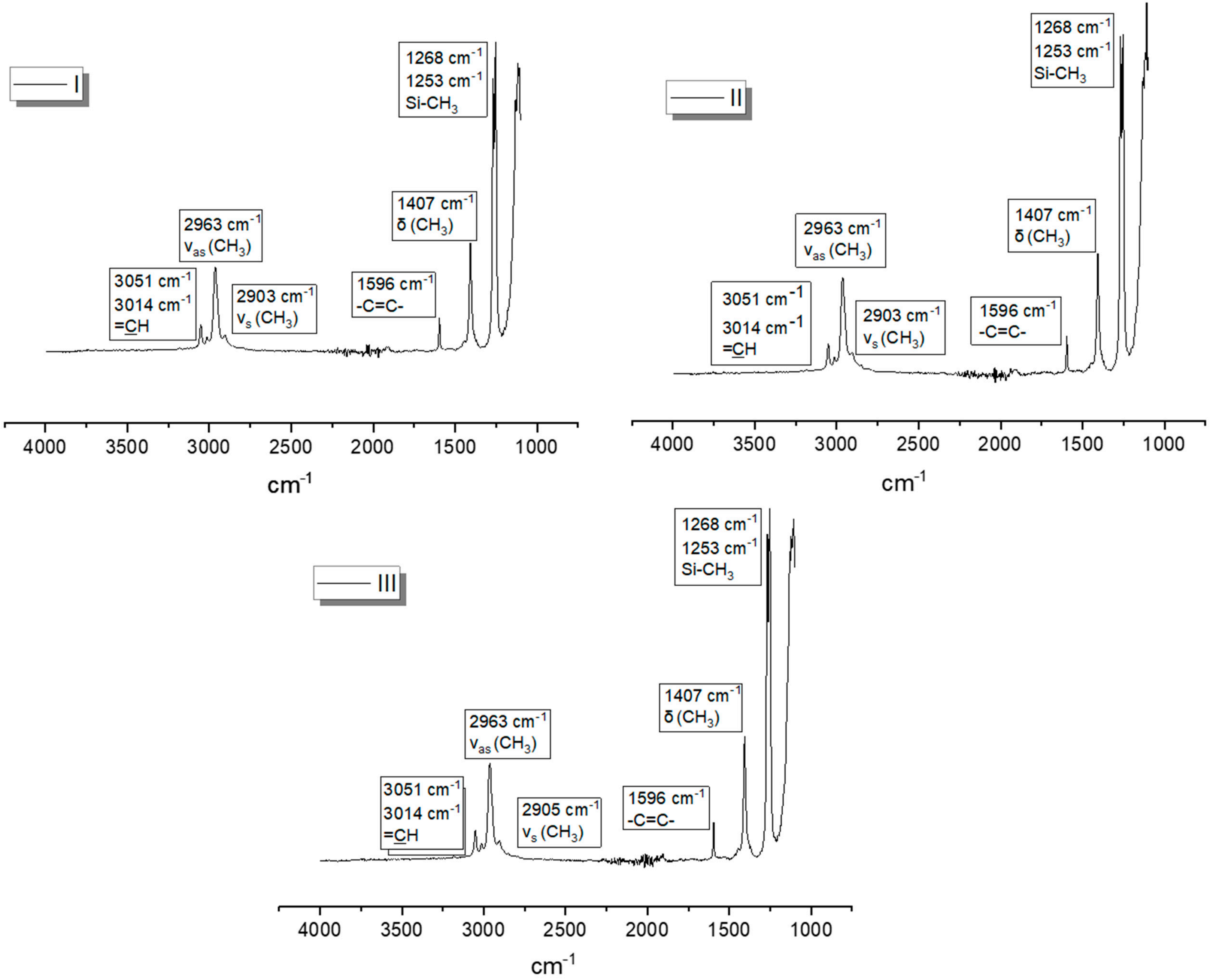

Figure S2. IR spectra of polysodiumoxy(methyl)siloxanes obtained in ethanol and blocked under blocking conditions: I - 1, II - 2, III – 3.
